# Evaluating ECAMM-based training efficacy for malaria microscopists in Hunan Province, China

**DOI:** 10.3389/fpubh.2025.1598917

**Published:** 2025-06-06

**Authors:** Wei Ning, Shi-feng Zhuang, Lan Wen, Ming-zhong Xu, Miao-miao Wang, Fang Peng, Fei-fan Huang, Bin Tian

**Affiliations:** ^1^Department of Clinical Laboratory, Changsha Municipal Center for Disease Control and Prevention, Changsha, China; ^2^Department of Endemic and Parasitic Diseases Control, Hunan Center for Disease Control and Prevention, Changsha, China; ^3^Xiangya School of Basic Medical Sciences, Central South University, Changsha, China

**Keywords:** ECAMM, microscopist, malaria, competency, training course

## Abstract

**Background:**

The rapid and accurate diagnosis of malaria has contributed to China’s remarkable achievement in malaria elimination. In the post-elimination phase, microscopic examination remains crucial for preventing the reintroduction of imported malaria. This study developed and validated a 4-day external competency assessment training program for malaria microscopists based on the External Competency Assessment for Malaria Microscopists (ECAMM) framework, to enhance diagnostic proficiency among laboratory personnel in public health institutions.

**Methods:**

Course design, blood smear selection, evaluation methods, and scoring criteria strictly adhered to WHO guidelines. Participants underwent one pre-training test and four post-training assessments involving 48 blood smears to evaluate diagnostic accuracy in qualitative identification and species differentiation. A comprehensive analysis was conducted on training needs, skill improvement trajectories, diagnostic error patterns, competency predictors, and participant satisfaction. 24 participants from public health institutions across 14 prefecture-level jurisdictions in Hunan Province completed the program with heterogeneous performance outcomes.

**Results:**

75% of participants had received no formal malaria microscopy training in the preceding 5 years. Post-training assessments demonstrated significant improvements: negative smear recognition accuracy increased by 29.86%, positive smear identification by 25.52%, and species differentiation accuracy by 48.96%. The predominant diagnostic error was interspecies confusion, notably the misidentification of *Plasmodium ovale* (*P. ovale*) as *Plasmodium malariae* (*P. malariae*)(20.8% of errors). Trainee competency showed no significant correlation with age, gender, or educational background (*p* > 0.05). Participant satisfaction ratings consistently reached “very satisfied” with almost all training components.

**Conclusion:**

This external capacity assessment training program effectively enhanced short-term malaria microscopy proficiency. We propose institutionalizing this model as a national certification program to maintain diagnostic competency through regular training and quality monitoring, particularly targeting primary healthcare facilities at the township level.

## 1 Introduction

Despite a significant reduction in malaria-endemic countries from 108 in 2000 to 83 in 2023, malaria remains a life-threatening global health crisis, with an estimated 263 million cases reported in 2023 (1, 2). While this progress demonstrates the feasibility of elimination, the persistent burden underscores the urgent challenge of preventing re-establishment in targeted regions—particularly through imported cases that threaten hard-won achievements. China’s malaria-free certification (3), a landmark accomplishment, exemplifies both the success and ongoing vulnerability of elimination efforts. Between 2013 and 2022, China reported over 24,500 imported malaria cases (4, 5), with sustained risk of local transmission resurgence due to the presence of competent *Anopheles* mosquito vectors (6, 7). In this fragile post-elimination phase, maintaining high-level surveillance and diagnostic capacity is critical for rapid case detection and containment—a lesson with profound implications for global malaria elimination sustainability.

Microscopic blood smear examination, the gold standard for malaria diagnosis, serves as the cornerstone of China’s surveillance system. Alarmingly, declining opportunities for practical experience have led to a shortage of skilled mcroscopists (6–8), directly threatening public health systems through potential diagnostic errors or delayed case detection. This crisis necessitates robust competency assessment programs (9, 10).

The WHO’s External Competency Assessment of Malaria Microscopists (ECAMM) framework has been widely adopted due to its standardized design, practical implementation, and global acceptance. It employs standardized test panels comprising 20 negative slides, 20 positive slides (single/mixed infections at defined parasite densities), and 16 *Plasmodium falciparum* (P. *falciparum*)-positive thick/thin blood smears for quantification (9). All slides are verified by ECAMM Level 1-certified experts and molecular diagnostics. Participants must complete examinations within 10 min per slide (11, 12), simulating real-world diagnostic pressures (6, 8, 9).

Building on this framework, we designed a novel 4-day training program specifically tailored for semi-skilled personnel in post-elimination settings—a critical unmet need in malaria surveillance. Traditional malaria microscopy training programs focus on pre-elimination high-transmission scenarios, requiring a long time for parasite quantification mastery. Our condensed 4-day framework prioritizes diagnostic accuracy over quantification—a strategic adaptation reflecting post-elimination epidemiology where rapid case confirmation outweighs density measurement needs. Our pilot program in Hunan Province featured 24 participants (75% lacking formal microscopy training in the preceding 5 years since 2019), with curriculum components:

**Pre-assessment:**.
  - 135-min baseline skill evaluation.
  - 30-min expert feedback.
**Theoretical training:** Four didactic lectures (420 min total).
**Competency verification:** Four 105-min standardized assessments.
**Stage evaluation:**.
  - Test result announcements.
  - Incorrect slide review.
  - On-site Q&A sessions (240 min total).
**Course evaluation:** Structured participant surveys.

To evaluate the training effectiveness and provide a basis for its continuous improvement, a comprehensive analysis was carried out covering participant’s needs, diagnostic skill progression, error pattern analysis, factors influencing blood smear interpretation skills, and satisfaction surveys. This short-term external competency assessment training program effectively enhanced proficiency in malaria microscopy. Through standardized training and periodic quality assessments, it can effectively prevent secondary transmission risks of imported malaria in elimination settings, offering a replicable China experience for global malaria elimination efforts. The comprehensive analysis of error patterns and skill progression metrics provides a critical foundation for refining training frequency and content prioritization in future iterations, particularly for regions transitioning to post-elimination surveillance.

## 2 Methods

### 2.1 Participant recruitment

The training program was organized by the Hunan Provincial Center for Disease Control and Prevention (CDC). Participants comprised malaria microscopists from CDC-affiliated institutions across 14 municipal cities and autonomous prefectures in Hunan Province, with each institution dispatching 1–2 personnel responsible for malaria microscopy.

### 2.2 Equipment and material preparation

The following equipment and materials were prepared: 24 high-resolution bright-field microscopes, one digital camera-equipped microscope system (Leica DM2000), a computerized imaging station (computer with printer), and Giemsa-stained thick/thin blood smears. Before the slide-reading proficiency assessment, all blood smears underwent rigorous validation through consensus review by three ECAMM Level 1-certified experts and confirmatory nucleic acid amplification testing. The detailed composition of the blood smears is presented in Table 1.

**Table 1:** Blood smear composition.

Test round	Parasitemia (p/*μl*)	Number of positive blood smears				
		*pf*	*pv*	*pm*	*po*	NMPS
Pre (*n* = 12)	<200	0	0	0	0	4
	201–500	1	1	0	1	
	501–2,000	1	1	1	1	
	2,001–5,000	1	0	0	0	
	Sum	3	2	1	2	4
r1 (*n* = 9)	<200	0	0	0	1	4
	201–500	0	0	0	0	
	501–2,000	1	1	1	0	
	2,001–5,000	1	0	0	0	
	Sum	2	1	1	1	4
r2 (*n* = 9)	<200	1	0	0	0	5
	201–500	1	0	0	1	
	501–2,000	0	0	0	0	
	2,001–5,000	0	0	0	1	
	Sum	2	0	0	2	5
r3 (*n* = 9)	<200	1	0	0	0	4
	201–500	1	1	1	0	
	501–2,000	0	0	0	1	
	2,001–5,000	0	0	0	0	
	Sum	2	1	1	1	4
r4 (*n* = 9)	<200	0	0	0	0	5
	201–500	2	0	0	1	
	501–2,000	0	0	0	1	
	2,001–5,000	0	0	0	0	
	Sum	2	0	0	2	5

pf, plasmodium falciparum; pv, plasmodium vivax; pm, plasmodium malariae; po, plasmodium ovale; NMPs, none malaria parasites.

Furthermore, prepare both electronic and paper materials, including the WeChat application, to accommodate participants’ theoretical tests, questionnaires, training materials, and feedback collection. Additionally, ensure that 24 bottles of immersion oil, sufficient lens cleaning paper, writing instruments, and requisite office and statistical analysis software are available.

### 2.3 Course setup and implementation

The 4-day training program integrated ECAMM core modules through adapted modalities: didactic lectures, slide-reading evaluations, group reviews of misdiagnosed slides, microscope maintenance practicums, theoretical knowledge quizzes, interactive feedback sessions, and structured participant surveys.

Critical ECAMM components were strategically integrated into specific theoretical training phases: blood elements differentiation and pseudo-parasites/artefacts identification were embedded within r2-stage theoretical training through analysis of prepared blood slides. The r3-stage theoretical training includes parasite quantification. Quality assurance principles were incorporated into r4-stage curriculum modules. Notably, parasite quantification training was provided but not formally assessed, as 75% of participants lacked prior microscopy training (past 5 years)—baseline skill levels precluded quantification assessment (requires advanced proficiency).

**Figure 1:**
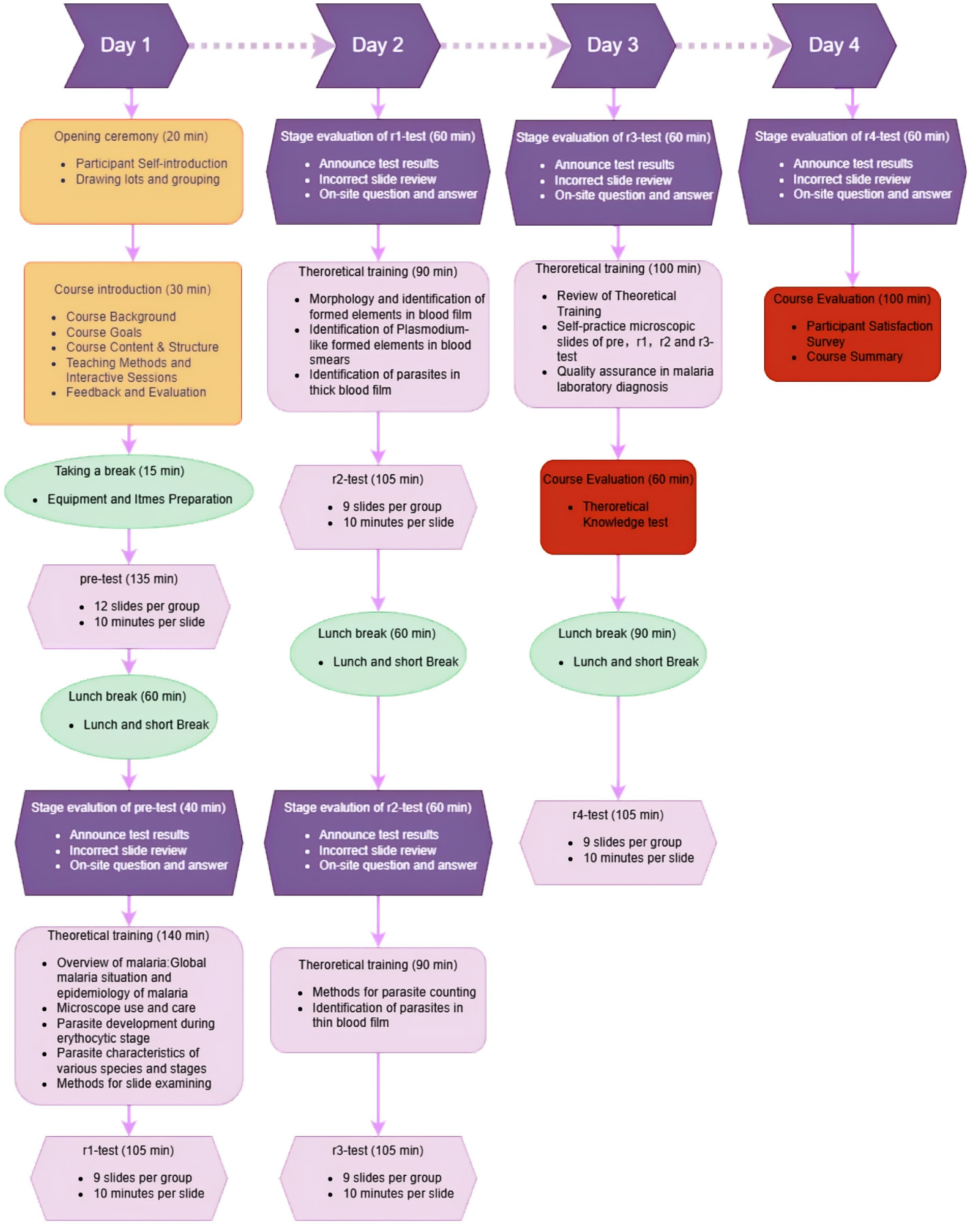
Course setup and implementation flowchart.

During group reviews, slides achieving ≥30% participant consensus for interpretive errors underwent detailed review via a camera-integrated live tracking microscopy system, enabling trainer-guided coordinated analysis with real-time annotation capabilities. For slide-reading assessments, participants were divided into 12-member cohorts to conduct microscopic evaluations concurrently. Each microscopist was required to independently examine the slides and submit diagnostic interpretations within a strictly enforced 10-min examination window, with inter-participant communication prohibited. The standardized assessment protocol and procedural workflow are detailed in Figure 1.

### 2.4 Data collection and analysis

Data collected from paper-based tests, questionnaires, and WeChat-based applications were directly entered into Microsoft Excel (Home and Student Edition 2021) on-site. Subsequently, these data were imported into IBM SPSS Statistics 28.0 for statistical analysis. Non-parametric comparisons between pre-training assessments and four post-training evaluations were conducted using Wilcox-test, with competence scores expressed as median values and 95% confidence intervals (95% CI). To verify the factors influencing diagnostic competency, Kendall’s tau-*b* correlation coefficients were computed to examine relationships between participants’ background information (n = 24) and diagnostic performance metrics (pre-test scores, post-training test series). The statistical significance threshold was set at *α* = 0.05 (two-tailed). The differences were considered significant when *p* < 0.05 and non-significant when *p* ≥ 0.05. The tables featured in this paper were generated using SPSS. For data visualization, the SPSS-processed data were uploaded to the CNSknowall platform,^1^ a versatile online tool for data analysis and visualization.

## 3 Results

### 3.1 Basic information of training participants

The cohort comprised 24 public health professionals from CDC institutions across 14 cities and autonomous prefectures in Hunan Province (Table 2). Municipal-level CDCs accounted for 79.17% (19/24) of participants, while 20.83% (5/24) represented county-level institutions. Participants ranged in age from 24 to 50 years (mean ± SD: 31.0 ± 6.2 years), with a female predominance (male: female = 0.33:1). Educational backgrounds revealed a significant proportion of participants (83.34%) held a bachelor’s degree or higher. Specifically, 58.33% (14/24) specialized in Medical Laboratory Science, 8.33% (2/24) in Public Health, and 29.17%(7/24) had a background in Public Health Laboratory Science. Regarding their job positions, the majority of these participants are engaged in clinical laboratory testing, physical and chemical testing, and disease prevention and control. Among them, the clinical laboratory testing position accounts for 83.33%. However, since malaria has been eliminated in China, laboratory staff rarely have the opportunity to actually encounter malaria cases. Professional titles analysis showed 83.33% (20/24) held junior-to-intermediate technical positions. Regarding training history, 75.00% (18/24) had received no formal theoretical or practical malaria microscopy training in the preceding 5 years. Practical experience gaps were evident, 45.83% (11/24) reported never having examined malaria blood smears during this period, and 41.67% (10/24) had handled ≤5 malaria cases annually. Notably, 62.50% (15/24) lacked hands-on experience in *Plasmodium* morphological identification, indicating a potential gap in practical diagnostic competency.

**Table 2:** Basic information of training participants.

Project	Classification	Participants *n* = 24 (%)	
Age	≤24 years	1	4.17
	>24 ~ 30 years	11	45.83
	>30 ~ 40 years	10	41.67
	>40 years	2	8.33
Unit level	Municipal level	19	79.20
	County level	5	20.80
Gender	Male	6	25.00
	Female	18	75.00
Educational background	Associate degree	4	16.67
	Bachelor’s degree	16	66.66
	Master’s degree	4	16.67
Professional background	Public health	2	8.33
	Basic medical sciences	1	4.17
	Public health laboratory science	7	29.17
	Medical laboratory science	14	58.33
Job position	Clinical laboratory testing	20	83.33
	Physical and chemical testing	2	8.33
	Disease Prevention and Control	1	4.17
	Microbial testing	1	4.17
Professional title	Without	2	8.33
	Junior	10	41.67
	Intermediate	10	41.67
	Senior	2	8.33
Received theoretical or practical training in the past 5 years	Without theoretical and practical training	18	75.00
	Only theoretical training	2	8.33
	Both theoretical and practical training	4	16.67
Frequency of training received in past 5 years	Never	18	75.00
	Once	4	16.67
	2 to 5 times	2	8.33
Annual slides reading over the past 5 years(slides/year)	Never	11	45.83
	1 to 5	10	41.67
	6 to 10	2	8.33
	over10	1	4.17
Time elapsed since the last smear reading	1 week	2	8.33
	1 month	6	25.00
	1–3 months	3	12.50
	6 to 12 months	5	20.83
	Over 12 months	8	33.34
Observed the microscopic morphology of four types of parasite (Yes or No)	Yes	9	37.50
	No	15	62.50
Observed the microscopic morphology of all parasite stages (Yes or No)	Yes	3	12.50
	No	21	87.50

### 3.2 Blood smear diagnostic competency

Pre-training assessments revealed substantial variability in diagnostic accuracy for qualitative assessment of negative blood smears, with values ranging from 0 to 100% (mean: 43.75%; 95% CI: 31.95 to 56.73) among the 24 participants. For positive blood smears, the accuracy was similarly variable (range: 0 to 100%; mean: 63.02%; 95%CI: 54.61 to 72.36). Parasite species identification in positive smears demonstrated particularly low accuracy (range: 0 to 50%; mean: 14.58%; 95% CI: 9.00 to 21.15).

Post-training evaluations demonstrated progressive improvement. The first testing round (Round 1, r1) indicated negative smear accuracy increased to 54.17% (95% CI, 38.89 to 68.52). For positive smears, the accuracy improved to 87.50% (95% CI, 80.67 to 93.99), with species identification at 57.64% (95% CI, 50.69 to 65.22). Following the second theoretical training, Round 2 (r2) revealed significant variability in negative smears assessments (range: 0 to 100%; mean: 65.28%; 95% CI: 52.78 to 77.27). Positive smear accuracy further improved (mean: 91.67%; 95%CI: 86.51 to 96.49), though species identification accuracy declined to 44.44% (95% CI, 33.97 to 55.88). The third round of testing (r3) showed further improvement, with negative smear accuracy reaching 77.08% (95% CI, 67.39 to 86.95), positive smear accuracy stabilizing at 79.17% (95% CI, 73.73 to 84.99), and species identification recovering to 56.25% (95% CI, 48.33 to 65.27). During the fourth round (r4), participants achieved near-perfect negative smears interpretation (mean: 97.92%; 95% CI: 95.37 to 100), positive smears detection at 95.83% (95% CI, 89.29 to 100), and species identification matching positive smears accuracy (mean: 95.83%; 95% CI: 89.29 to 100).

**Figure 2:**
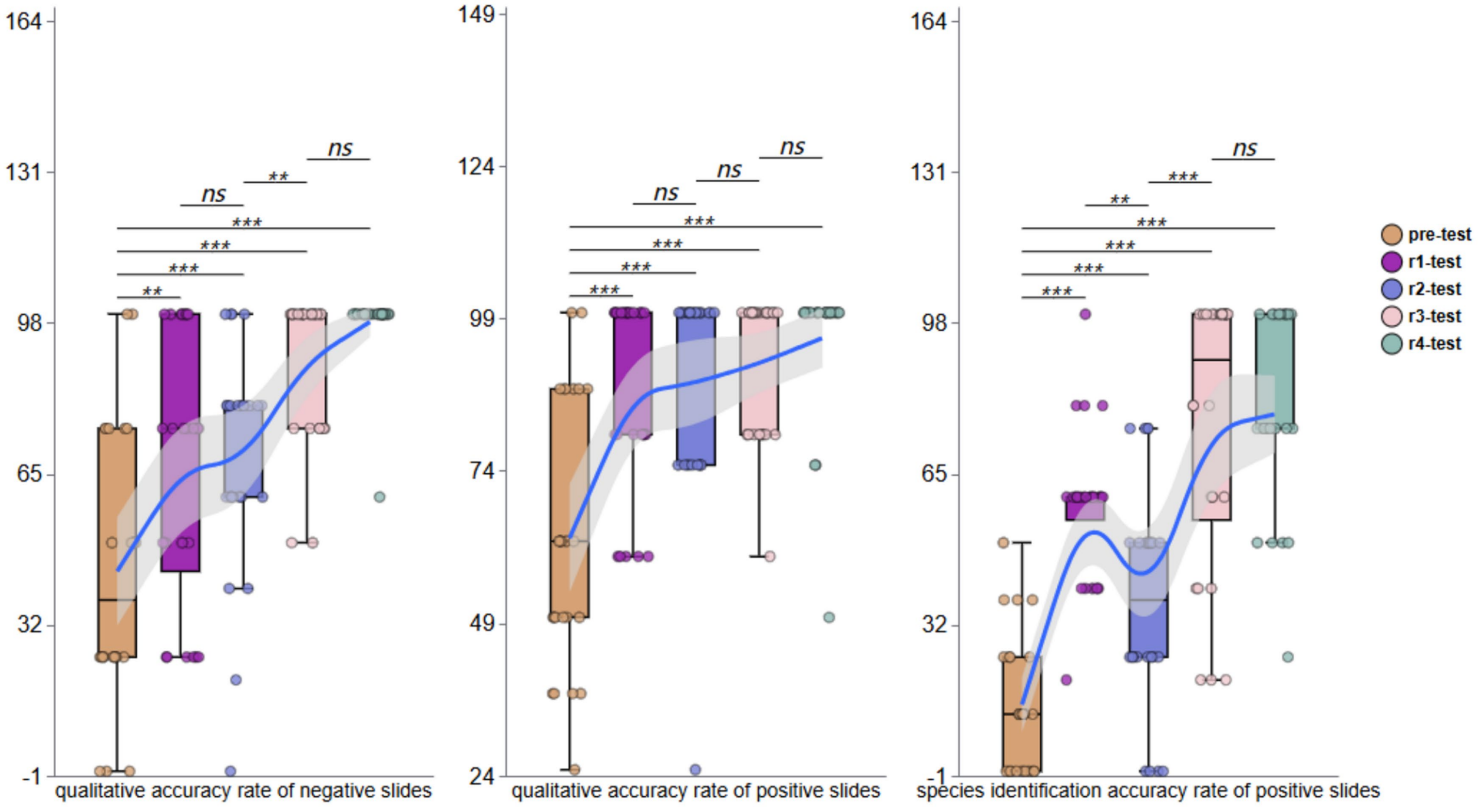
The qualitative and species identification accuracy of negative or positive smears.

Across all four training phases (r1-r4), overall mean accuracy was 73.61% (95% CI, 67.49–80.07) for negative smears, 88.54% (95% CI, 85.46 to 91.58) for positive smears, and 63.54% (95% CI, 58.06 to 69.02) for species identification. These results demonstrate a consistent upward trajectory in participants’ ability to qualitatively assess both negative and positive blood smears and identify parasite species, as visually summarized in Figure 2.

### 3.3 Correlation analysis

**Figure 3:**
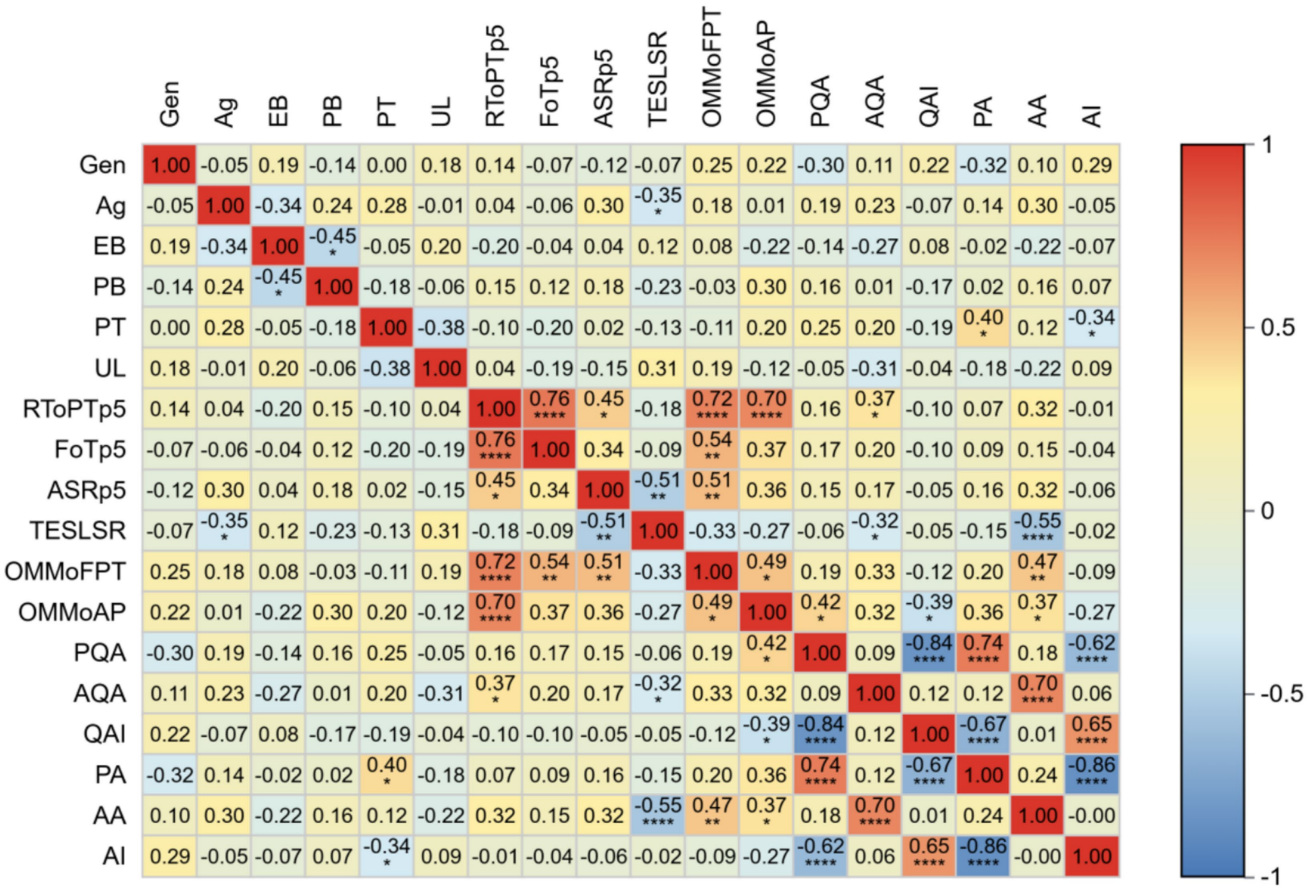
Heatmap of factors related to the improvement of participants’ abilities. Gen, Gender; Ag, Age; EB, Educational Background; PB, Professional Background; PT, Professional Title; UL, Unit Level; RToPTp5, Received Theoretical or Practical Training in the past 5 years; FoTp5, Frequency of Training received in past 5 years; ASRp5, Annual Slides Reading over the past 5 years; TESLSR, Time Elapsed Since the Last Seamer Reading; OMMoFPT, Observed the Microscopic Morphology of Four Types of Parasite; OMMoAP, Observed the Microscopic Morphology of All Parasite stages; PQA, Pre-test qualitative accuracy; AQA, after-training qualitative accuracy; QAI, Improvement in qualitative accuracy; PA,Pre-test species identification accuracy; AA, after-training species identification accuracy; AI, Improvement in species identification accuracy.

A correlation analysis was conducted on participants’ background information, pre-test scores, and performance across four post-training testing rounds. Results are presented in Figure 3, which displays pairwise correlation coefficients between variables of interest. The color intensity in the heatmap corresponds to the strength and direction of the correlation: warm colors indicate positive correlations, while cool colors denote negative correlations.

The analysis revealed a significant negative correlation between post-training malaria species-identification accuracy and time since last blood smear examination (*r* = −0.55, *p* < 0.0001), suggesting diagnostic competency decay over time. Furthermore, significant positive correlations were observed between post-training species identification accuracy and participants’ prior experience in identifying microscopic structures of parasites (*r* = 0.47, *p* < 0.01) and distinguishing parasite developmental stages (*r* = 0.37, *p* < 0.05). A moderate positive correlation was identified between post-training qualitative assessment accuracy and prior theoretical/practical training within the past 5 years (*r* = 0.37, *p* < 0.05).

Pre-training analysis demonstrated positive associations between qualitative assessment accuracy and microscopy experience (*r* = 0.42, *p* < 0.05), along with a correlation between pre-training species identification accuracy and professional titles (*r* = 0.42, *p* < 0.05).

Notably, no significant correlations were found between post-training performance and age, gender, educational background, or the number of blood smears examined over the preceding 5 years.

### 3.4 Error categories

During the four training rounds (r1-r4), 24 trainees evaluated 18 blood smears containing *non-malarial parasites* (nmps) generating 432 diagnostic entries. Among these, 348 (80.6%) entries were correctly identified as nmps. Misidentifications included 26 (6.0%) entries as *P. falciparum* (pf), 29 (6.7%) as *P. vivax* (pv), 18 (4.2%) as *P. ovale* (po), and 11 (2.5%) as *Plasmodium malariae* (pm).

**Figure 4:**
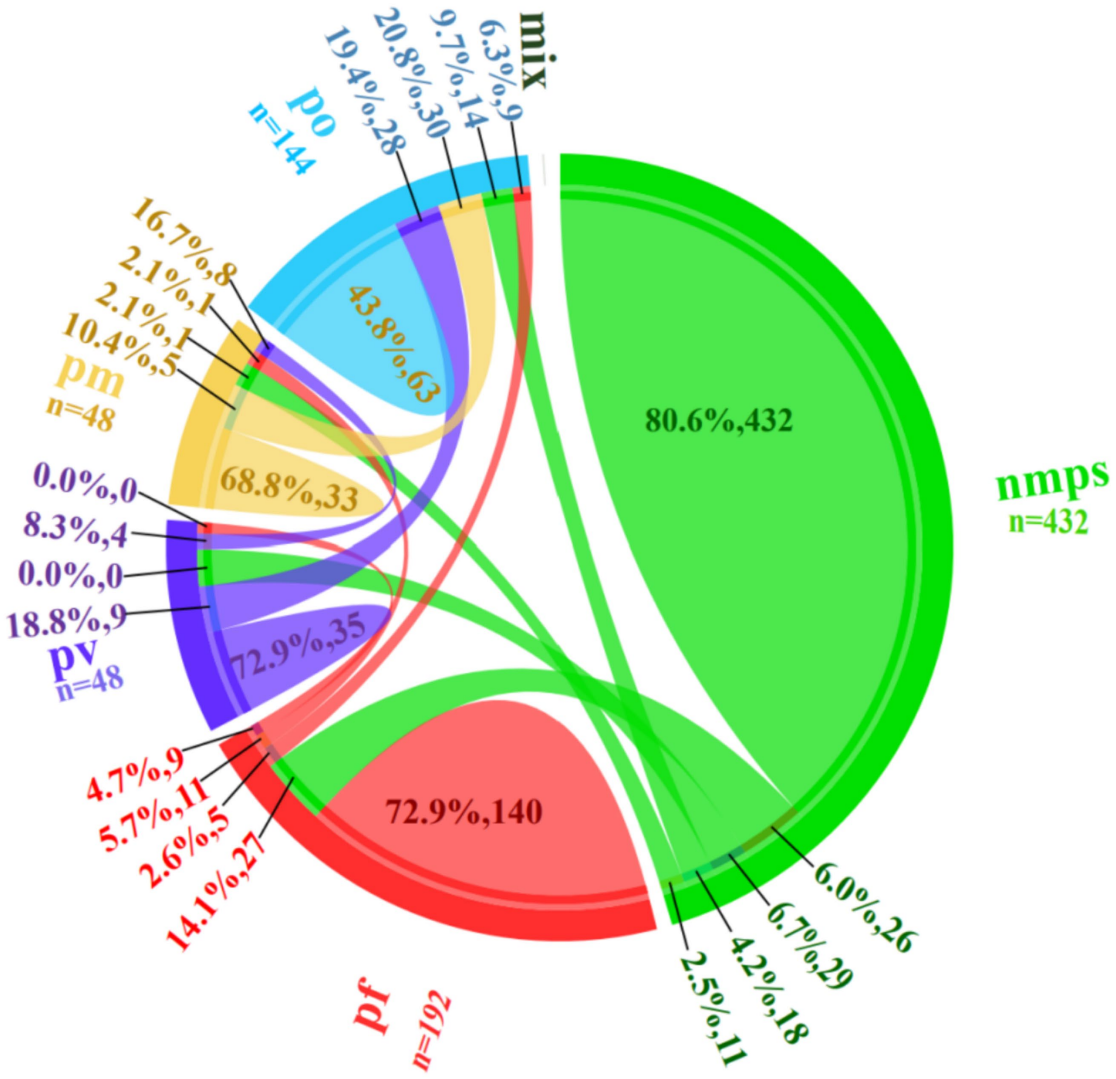
Error categories relationship diagram.

For 8 pf-positive blood smears, 192 responses were recorded, with 140 (72.9%) being accurate. Errors comprised 27 (14.1%) misclassified as nmps, 5 (2.6%) as po, 11 (5.7%) as pm, and 9 (4.7%) as pv. Analysis of 2 pv-positive blood smears yielded 48 responses, with 35 (72.9%) correct identifications. Errors included 9 (18.8%) misidentified as po and 4 (8.3%) as pm, with no pf misclassifications. Evaluation of 6 po-positive blood smears generated 144 responses, with 63 (43.8%) accurate diagnoses. Misclassifications were distributed as 28 (19.4%) as pv, 30 (20.8%) as pm, 9 (6.3%) as pf, and 14 (9.7%) as nmps. For 2 pm-positive blood smears, 48 responses were recorded, with 33 (68.8%) correct. Errors included 8 (16.7%) as pv, 5 (10.4%) as po, 1 (2.1%) as pf, and 1 (2.1%) as nmps (see Figure 4).

A detailed breakdown of the error categories and proportions is provided in Figure 3.

### 3.5 Motivation and satisfaction of participating

**Figure 5:**
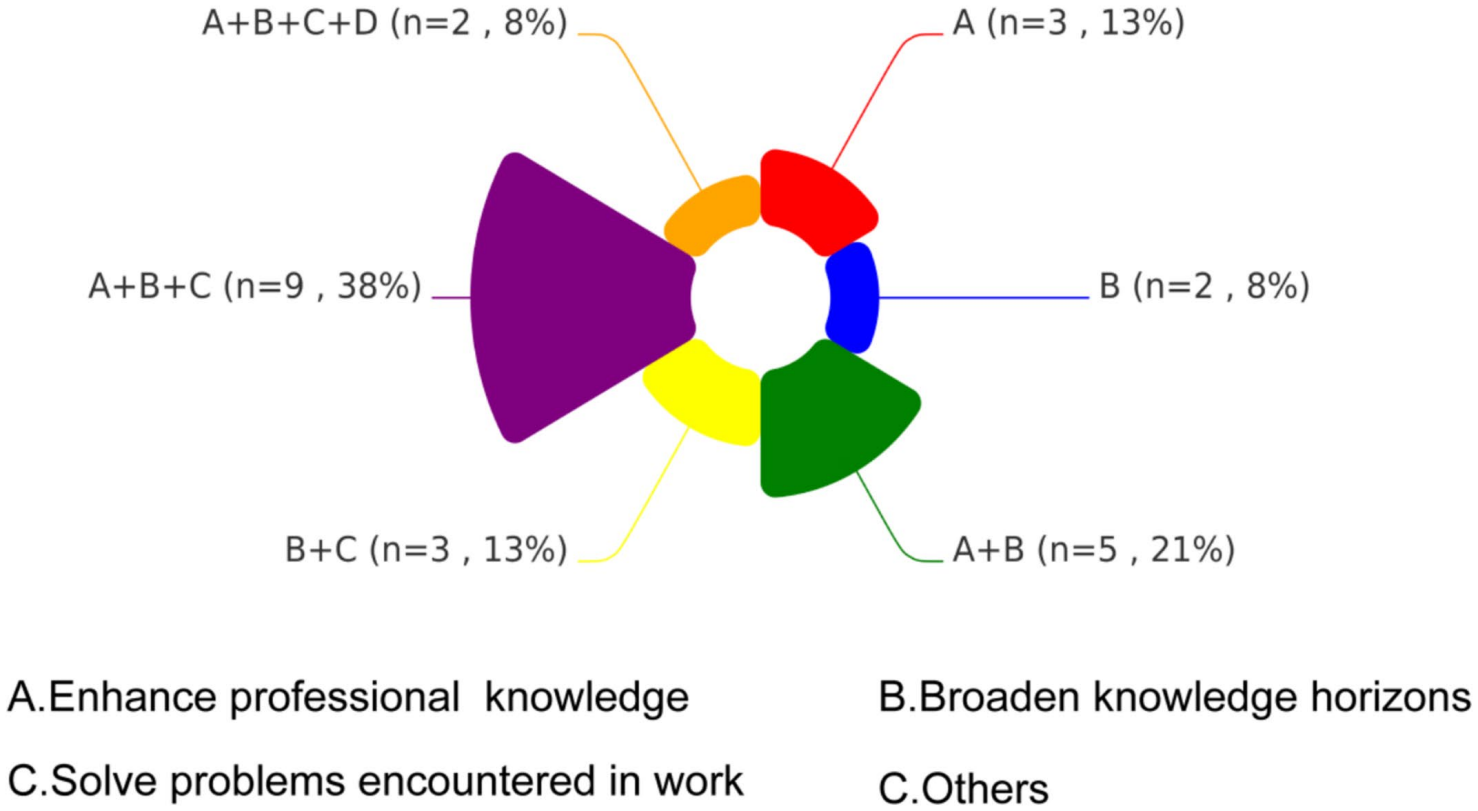
Distribution of learning motivation among training participants.

Figure 5 presents a chord diagram illustrating the distribution of learning motivations among participants, categorized into four domains: A (Enhance professional knowledge), B (Broaden knowledge horizons), C (Solve problems encountered in work), and D (Others). The largest proportion of participants (38%, *n* = 9) prioritized a combination of objectives A, B, and C., reflecting comprehensive learning needs. A dual focus on A and B was reported by 21% (*n* = 5), reflecting interest in both theoretical and practical knowledge expansion. The B + C combination was selected by 13% (*n* = 3), emphasizing a strong desire to apply new knowledge to practical challenges. An equivalent proportion of 13% (*n* = 3) focused solely on objective A. The least prevalent combination 8% (*n* = 2) encompassed all four domains.

**Figure 6:**
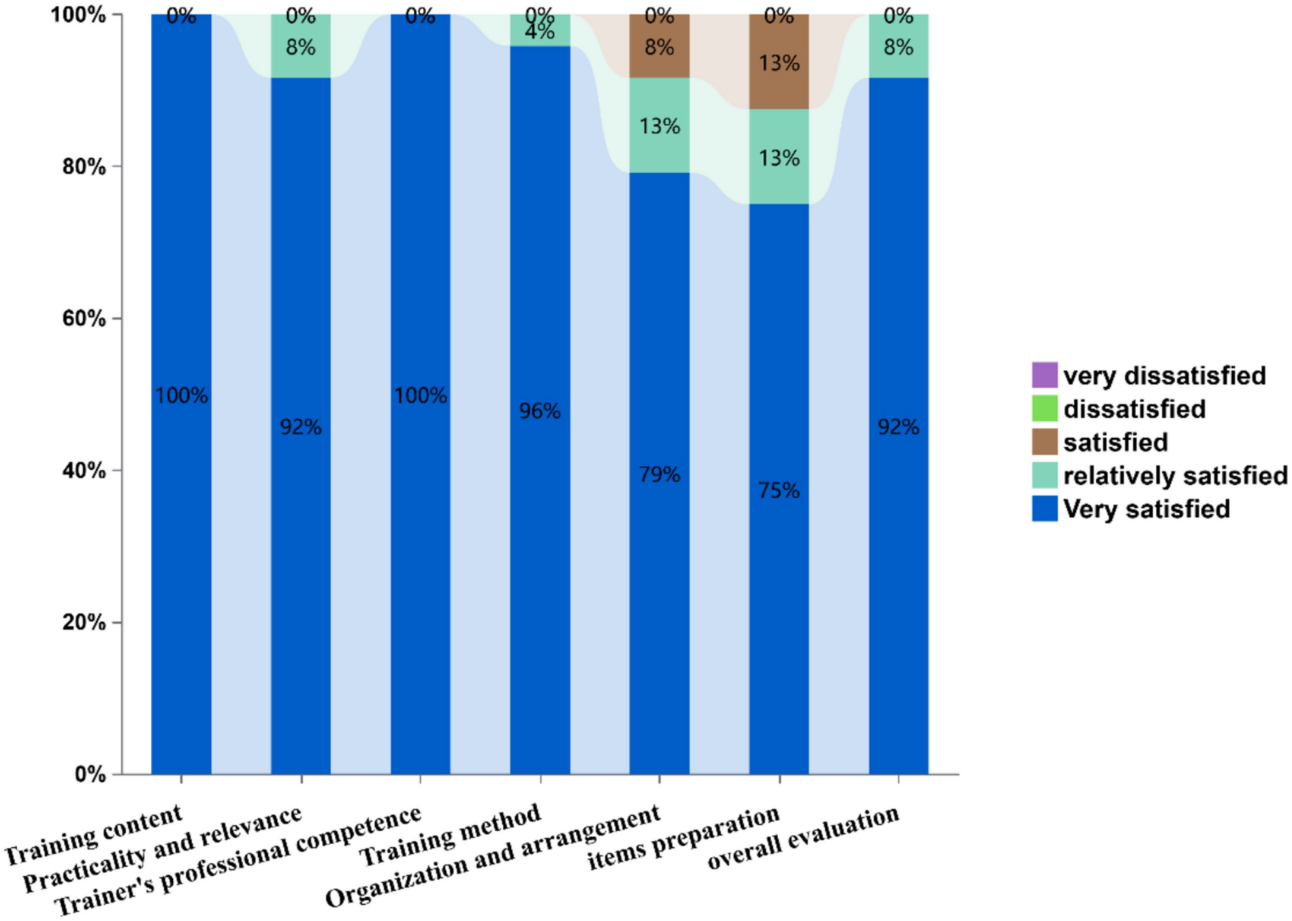
Participant satisfaction levels across various aspects of the training program.

Figure 6 displays training satisfaction metrics through a stacked bar chart. Evaluated dimensions included: training content, practicality and relevance, trainer’s professional competence, training method, organization and arrangement, items preparation, and overall evaluation. Satisfaction levels demonstrated exceptional performance in training content and trainer’s professional competence (100% “very satisfied”). Training method received 96% top ratings, while organization and arrangement showed 79% high satisfaction (13% “Relatively satisfied,” 8% “Satisfied”). Items preparation achieved 75% high satisfaction, and overall evaluation reached 92% top-tier endorsement. Notably, no negative evaluations (“dissatisfied” or below) were recorded across any parameters.

## 4 Discussion

Hunan Province—once a malaria-endemic region achieving zero indigenous transmission since 2011 (13)—now faces escalating imported malaria risks, with 542 cases reported during 2016–2021 (13). This epidemiological shift underscores the urgency of sustaining diagnostic vigilance. Additionally, there remains a gap in microscopic examination capabilities among provincial and municipal CDCs in China (14–16). This underscores the urgent need for capacity-building initiatives in malaria microscopy across all tiers of CDCs and healthcare facilities. In this study, we implemented the ECAMM framework to design a skill-enhancement program, evaluating its efficacy with 24 trainees representing CDCs from 14 municipal-level jurisdictions in Hunan Province.

The training significantly improved participants’ microscopic diagnostic competencies, demonstrating satisfactory training efficacy. Evaluations after four rounds of training revealed comprehensive accuracy enhancements: 29.86% increase in negative slide interpretation, 25.52% improvement in positive slide detection, 48.96% rise in species identification—performance gains exceeding regional benchmarks from Hubei Province (17.66% detection rate increase) and African studies (19.1–34.9% accuracy improvements) (17–19). These outcomes confirm the program’s alignment with global training efficacy patterns.

Phased assessments revealed differential skill progression. Following Day 1 theoretical training, round 1 testing showed immediate improvements: negative blood smear interpretation accuracy increased by 10.42%, positive smear detection rates improved by 24.48%, and species differentiation accuracy rose by 18.75%. This rapid progress correlated strongly with a structured curriculum covering parasite morphology across developmental stages, pathological alterations in infected red blood cells, and standardized protocols for thick/thin blood smears. These components enabled novices to establish foundational microscopy skills and conceptual frameworks. In round 2 testing, structured feedback and interactive smear review sessions featuring peer discussion and expert-led morphological clarification, effectively reinforcing parasite–host cell recognition. Supplementary modules on blood cell morphology differentiation, artifacts mimicking *Plasmodium*, and thick film parasite detection further contributed to competency development. Notably, round 2 species identification accuracy temporarily declined compared to round 1. This transitional phase reflects the cognitive overload during skill application, as trainees integrated theoretical knowledge into time-pressured decision-making—a adaptation phase in diagnostic skill acquisition. Furthermore, field-collected slides demonstrating atypical features compared to standardized teaching specimens also posed additional challenges for trainees. Competency rebounded in rounds 3–4, achieving 95.83% sensitivity and 97.92% specificity —surpassing WHO-recommended thresholds (9). These findings confirm the ECAMM-based training program effectively accelerates diagnostic proficiency in malaria microscopy among public health technicians.

Correlation analysis identified three key predictors of microscopy proficiency: shorter intervals since last slide examination, prior theoretical training and practical experience—all significantly enhanced post-training skills. It’s noteworthy that higher professional titles showed an inverse correlation with species identification improvement, contrasting with conventional patterns (20). This likely reflects senior staff’s limited malaria-specific given Hunan’s youth capacity-building priority via under-35 microscopy competitions (21, 22), which shaped the junior-dominated cohorts. The demographic factors (age, gender, educational background) and baseline slide experience showed no significant associations, aligning with muti-privince data (15, 16, 20), confirming curriculum quality—not trainee background—drives competency gains.

Retrospective analysis identified three interconnected error patterns in malaria microscopy: (1) False-positive identifications (negative smears misidentified as pv or pf) primarily due to morphological misinterpretation (platelets/Howell-Jolly’s body), artifact interference (slide deposition, pseudo-images, or stain precipitate), and technical limitations in fine-focus adjustment for debris differentiation. (2) Species misdifferentiation, notably pv-po confusion from misinterpreted erythrocyte margins (23) and parasite cytoplasm. Technical limitations may also contribute: smears prepared from anticoagulated blood can alter erythrocyte volume and color dots (24). (3) Size-related misclassification (20.8% po-pm errors) resulting from trainees’ inexperience in leveraging infected erythrocyte characteristics (e.g., Schüffner’s stippling) for differentiation, compounded by technical biases in parasite size estimation. Accurate identification of pf is not typically difficult unless for those inexperienced with blood smear examination (8). The main reason for misjudgment is a lack of in-depth understanding of stage-specific parasite morphology knowledge. Additionally, irregular medication adherence and immune-mediated atypical trophozoite presentations may obscure pf morphology (25). Fundamentally, most participants lacked prior formal microscopy training or hands-on experience in malaria microscopy, consistent with their baseline performance, this highlights the critical need for standardized competency-based training programs targeting subprovincial diagnostician’s level.

Analysis of trainees’ motivations revealed high regard for the training opportunity and strong enthusiasm for skill development. The strong enthusiasm driven by two synergistic factors: (1) China’s performance evaluation system where provincial CDCs annually assess subprovincial malaria microscopy capabilities, directly impacting institutional funding allocations—a key driver for engagement. WHO emphasizes such intrinsic motivation as critical for achieving microscopy excellence (9). (2) Participants had an intrinsic motivation to address field challenges and aware of the skill gaps identified in pre-training surveys. This contrasts sharply with Angola’s failed training model (26), where poorly structured curricula and inadequate incentives led to participants attrition, underscoring our program’s success in aligning institutional priorities with learner needs. Participants universally rated the program’s pedagogical structure as excellent, but flagged organizational gaps: insufficient consumables (e.g., lens cleaning paper) and subpar microscope maintenance reduced management satisfaction scores. These structural deficits, quantified through post-training feedback, directly inform the recommendation for standardized pre-session resource audits to bridge competency-development and operational support.

Despite the positive outcomes, our research encountered certain constraints. Training 24 participants exceeded ECAMM’s recommended cohort size of 12 (12), increasing logistical challenges, future iterations will adopt smaller cohorts and phased skill modules to preserve instructional quality. The absence of pf-pv/po mixed-infection—a limitation stemming from non-endemic specimen scarcity—necessitates establishing a national reference blood smear bank, as demonstrated by India’s successful repository model (27). Although the condensed 4-day program achieved WHO-recommended proficiency thresholds, its long-term efficacy requires validation through mandated 6-month competency reassessments, particularly for township-level staff engaged in frontline diagnosis. Few organizations provide such services in China, and access to refresher courses and training is challenging. In the next phase, we will propose a tripartite “training-certification-monitoring” competency maintenance framework.

In conclusion, novice technicians with no prior malaria microscopy experience can achieve significant diagnostic competency improvements through our structured training program. Sustaining these capabilities requires the implementation of periodic refresher courses integrated with routine external competency-based training to ensure sustained proficiency and quality assurance. The curriculum outlined in this study could serve as the foundation for a nationally endorsed certification program, enabling standardized training and proficiency monitoring of microscopists across all healthcare tiers, with prioritized implementation in township-level facilities.

## Glossary

ECAMM: External Competence Assessment of Malaria Microscopists
CDC: Center for Disease Control and Prevention
Pv: *P. vivax*
Pf: *Plasmodium* falciparum
Po: *Plasmodium* ovale
Pm: *Plasmodium malariae*
nmps: non-malarial parasites
Gen: Gender
Ag: Age
EB: Educational Background
PB: Professional Background
PT: Professional Title
UL: Unit Level
RToPTp5: Received Theoretical or Practical Training in the past 5 years
FoTp5: Frequency of Training received in past 5 years
ASRp5: Annual Slides Reading over the past 5 years
TESLSR: Time Elapsed Since the Last Seamer Reading
OMMoFPT: Observed the Microscopic Morphology of Four Types of Parasite
OMMoAP: Observed the Microscopic Morphology of All Parasite stages
PQA: Pre-test qualitative accuracy
AQA: after-training qualitative accuracy
QAI: Improvement in qualitative accuracy
PA: Pre-test species identification accuracy
AA: after-training species identification accuracy
AI: Improvement in species identification accuracy

## Data Availability

The original contributions presented in the study are included in the article/supplementary material, further inquiries can be directed to the corresponding author.

## Footnotes

^1^ https://cnsknowall.com

## References

[1] World Health Organization. World malaria report 2023. Geneva: World Health Organization (2023).

[2] World Health Organization. World malaria report 2024: Addressing inequity in the global malaria response. Geneva: World Health Organization (2024).

[3] Burki T. Triumph in China as it is certified malaria-free by WHO. Lancet Infect Dis. (2021) 21:1220–1. doi: 10.1016/S1473-3099(21)00491-6, PMID: 34450073

[4] Zhu Y Restrepo AC Wang H-B Mills DJ Liang R-R Liu Z-B. Malaria cases in China acquired through international travel, 2013-2022. J Travel Med. (2024) 31:56. doi: 10.1093/jtm/taae056, PMID: 38591791 PMC11646087

[5] Zhou XN. China declared malaria-free: a milestone in the world malaria eradication and Chinese public health. Infect Dis Poverty. (2021) 10:98. doi: 10.1186/s40249-021-00882-9, PMID: 34253259 PMC8276478

[6] Yan H Li M Xia ZG Yin JH. Competency of malaria laboratory diagnosis at national and provincial levels at the beginning of malaria post-elimination phase, China. Malar J. (2024) 23:58. doi: 10.1186/s12936-024-04883-5, PMID: 38408991 PMC10898020

[7] Yin J Li M Yan H Zhou S Xia Z. Laboratory diagnosis for malaria in the elimination phase in China: efforts and challenges. Front Med. (2022) 16:10–6. doi: 10.1007/s11684-021-0889-7, PMID: 35226298 PMC8883009

[8] Li M Zhou H Yan H Yin J Feng X Xia Z. Analysis on external competency assessment for malaria microscopists in China. Malar J. (2019) 18:366. doi: 10.1186/s12936-019-2996-3, PMID: 31727074 PMC6857338

[9] World Health Organization. Malaria microscopy quality assurance manual, version. 2nd ed. Geneva: World Health Organization (2016).

[10] Dong Y Deng Y Xu Y Chen M Wei C Zhang C. Analysis of initial laboratory diagnosis of malaria and its accuracy compared with re-testing from 2013 to 2018 in Yunnan Province, China. Malar J. (2020) 19:409. doi: 10.1186/s12936-020-03477-1, PMID: 33183296 PMC7664069

[11] Horning MP Delahunt CB Bachman CM Luchavez J Luna C Hu L. Performance of a fully-automated system on a WHO malaria microscopy evaluation slide set. Malar J. (2021) 20:110. doi: 10.1186/s12936-021-03631-3, PMID: 33632222 PMC7905596

[12] World Health Organization. Regional Office for South-East a: Workshop on external competence assessment and national competence assessment for malaria microscopists (ECAMM and NCAMM): Report of meeting, New Delhi, India. New Delhi: World Health Organization (2021).

[13] Zhuo HE Shifeng ZHUANG Zhengxiang LI Xiaolei LONG Guoqing WANG Jinping P. Epidemic characteristics of malaria in Hunan Province, 2016-2021. Pract Prevent Med. (2023) 30:818–22.

[14] Yin J Zhang L Feng J Zhou S Xia Z. Malaria diagnosis and verification-China, 2017-2018. China CDC Wkly. (2020) 2:285–8. doi: 10.46234/ccdcw2020.073, PMID: 34594640 PMC8422170

[15] Ding G Zhu G Cao C Miao P Cao Y Wang W. The challenge of maintaining microscopist capacity at basic levels for malaria elimination in Jiangsu Province, China. BMC Public Health. (2018) 18:489. doi: 10.1186/s12889-018-5307-y, PMID: 29650008 PMC5898017

[16] Fei L Shuang Z Yi Y Shan-Shan L Yan T Jing-Ru X. Assessment of malaria microscopy competency at primary health institutions in the Chongqing municipality. Front Med. (2021) 8:602442. doi: 10.3389/fmed.2021.602442, PMID: 33791321 PMC8005570

[17] Worges M Whitehurst N Saye R Ndiaye D Yamo E Yukich J. Performance outcomes from Africa-based malaria diagnostic competency assessment courses. Am J Trop Med Hyg. (2019) 100:851–60. doi: 10.4269/ajtmh.18-0361, PMID: 30793691 PMC6447135

[18] Kiggundu M Nsobya SL Kamya MR Filler S Nasr S Dorsey G. Evaluation of a comprehensive refresher training program in malaria microscopy covering four districts of Uganda. Am J Trop Med Hyg. (2011) 84:820–4. doi: 10.4269/ajtmh.2011.10-0597, PMID: 21540396 PMC3083754

[19] Aiyenigba B Ojo A Aisiri A Uzim J Adeusi O Mwenesi H. Immediate assessment of performance of medical laboratory scientists following a 10-day malaria microscopy training programme in Nigeria. Glob Health Res Policy. (2017) 2:32. doi: 10.1186/s41256-017-0051-x, PMID: 29202100 PMC5683359

[20] Suhua LI Penghui JI Zhiquan HE Ruimin ZHOU Dan WANG Dan QIAN. Hongwei Z: assessment on malaria microscopic examination ability at the city level in Henan province in 2023. Modern Dis Control Prevent. (2023) 2:95–9. doi: 10.13515/j.cnki.hnjpm.1006-8414

[21] Ze-Lin Z Yu-Wan H Tian T Chui-Zhao X Hong T Shuai H. Assessment of ability of professionals in parasitic disease control and prevention techniques in China, 2017. Zhongguo Xue Xi Chong Bing Fang Zhi Za Zhi. (2018) 30:518–22. doi: 10.16250/j.32.1374.2018112, PMID: 30567022

[22] Zhang S-S Xia Z-G Yin J-H Yan H Zhou S-S Li S-Z. Analysis report of the national technique competition for diagnosis of parasitic diseases in 2012: I. Capability analysis of plasmodium detection. Zhongguo Ji Sheng Chong Xue Yu Ji Sheng Chong Bing Za Zhi. (2013) 31:131–4. PMID: 24809195

[23] Kotepui M Masangkay FR Kotepui KU De Jesus MG. Misidentification of plasmodium ovale as plasmodium vivax malaria by a microscopic method: a meta-analysis of confirmed *P. ovale* cases. Sci Rep. (2020) 10:21807. doi: 10.1038/s41598-020-78691-7, PMID: 33311528 PMC7733466

[24] Tegegne B Ejigu K Alemu G Fetene Y Endaylalu K Melese M. Performance of malaria microscopy external quality assessment and networking among health facilities in West Amhara region, Ethiopia. BMC Infect Dis. (2020) 20:355. doi: 10.1186/s12879-020-05077-5, PMID: 32429860 PMC7236141

[25] Suwanarusk R Cooke BM Dondorp AM Silamut K Sattabongkot J White NJ. The deformability of red blood cells parasitized by plasmodium falciparum and *P. vivax*. J Infect Dis. (2004) 189:190–4. doi: 10.1086/380468, PMID: 14722882

[26] Moura S Fançony C Mirante C Neves M Bernardino L Fortes F. Impact of a training course on the quality of malaria diagnosis by microscopy in Angola. Malar J. (2014) 13:437. doi: 10.1186/1475-2875-13-437, PMID: 25406586 PMC4247670

[27] Nema S Srivastava B Ahmad N Sharma S Anvikar AR Rahi M. Malaria slide Bank to strengthen and improve the quality of malaria diagnosis: a National Slide Repository in India. Am J Trop Med Hyg. (2024) 110:921–4. doi: 10.4269/ajtmh.23-0429, PMID: 38579702 PMC11066356

